# Comprehensive diagnostics in a case of hereditary diffuse leukodystrophy with spheroids

**DOI:** 10.1186/s12883-015-0368-3

**Published:** 2015-07-04

**Authors:** Marie Meyer-Ohlendorf, Anne Braczynski, Omar Al-Qaisi, Florian Gessler, Saskia Biskup, Lutz Weise, Joachim P. Steinbach, Marlies Wagner, Michel Mittelbronn, Oliver Bähr

**Affiliations:** Department of Neurology, Goethe University Hospital, Frankfurt, Germany; Edinger Institute (Institute of Neurology), Goethe University Hospital, Frankfurt, Germany; Institute of Neuroradiology, Goethe University Hospital, Frankfurt, Germany; Department of Neurosurgery, Goethe University Hospital, Frankfurt, Germany; CeGaT GmbH, Tübingen, Germany; Hertie Institute for Clinical Brain Research, Tübingen, Germany; Dr. Senckenberg Institute of Neurooncology, University Hospital Frankfurt, Goethe University, Schleusenweg 2-16, 60528 Frankfurt, Germany

**Keywords:** HDLS, Leukoencephalopathy, MR spectroscopy, DWI, ADC, Restricted diffusion, CSF1R, Electron microscopy

## Abstract

**Background:**

Hereditary diffuse leukodystrophy with spheroids is a rare type of leukoencephalopathy. Mutations in the colony stimulating factor 1 receptor have recently been identified to be the cause of this microgliopathy. Clinical and radiological presentation can often misguide physicians during the diagnosis of patients with this underdiagnosed disease.

**Case presentation:**

We present a 29 year-old woman with a rapid course of hereditary diffuse leukodystrophy with spheroids. She mainly showed cognitive impairment and severe motor dysfunctions. Her MRI showed spotted and confluent hyperintensities of the white matter on T2-weighted images involving the corticospinal tract as well as the corpus callosum. Further, those lesions showed striking restricted diffusion. As this restricted diffusion in all areas showing signs of leukoencephalopathy was so impressive we searched Medline for these terms and got hereditary diffuse leukodystrophy with spheroids as one of the first results. After a comprehensive diagnostic workup and exclusion of other leukoencephalopathies, stereotactic biopsy and genetic testing confirmed the diagnosis.

**Conclusion:**

This case points out at two important features of hereditary diffuse leukodystrophy with spheroids being spotted and/or confluent leukoencephalopathy with areas of restricted diffusion. This might help to identify more patients with this underdiagnosed disease. Moreover, the rapid clinical course in our patient raises the question whether the relatively pronounced areas of restricted diffusion are indicative of a more acute progression of the disease.

## Background

Hereditary diffuse leukodystrophy with spheroids (HDLS) is a rare autosomal dominantly inherited disease that occurs in both familial and sporadic forms [1]. The median age of onset is in the fourth or fifth decade, and the reported range is from 8 to 78 years [2]. The disease inevitably leads to death within a few years after onset, however single cases with survival times of several decades have been described [3]. The clinical presentation can be variable including cognitive and behavioral changes, motor dysfunctions as well as seizures, ataxia and parkinsonism. Therefore clinical misdiagnoses such as multiple sclerosis (MS), frontotemporal dementia (FTD), corticobasal syndrome (CBS) or atypical cerebral autosomal-dominant arteriopathy with subcortical infarcts and leukoencephalopathy (CADASIL) are common in HDLS patient. Neuropathological features, which were first described in 1984 by Axelsson et al. comprise of widespread loss of myelin and the presence of neuroaxonal spheroids [3]. Typical changes seen in the MRI are predominantly bifrontal T2-hyperintense white matter lesions with deep and subcortical involvement [4, 5]. Also the corpus callosum and the corticospinal tracts are usually involved with lack of significant gray matter pathology [6]. Sundal et al. recently developed an HDLS MRI severity score showing that HDLS first manifests focally in the white matter and finally reaches a generalized confluent stage during disease progression [6]. Especially in early stages of the disease, MRI lesions can be misinterpreted as demyelinating or ischemic lesions or small vessel disease. Since clinical presentation is variable and also the changes in MRI are not specific, genetic testing or pathological examination is needed to confirm the diagnosis of HDLS. Just recently, mutations in the tyrosine kinase domain of the colony stimulating factor receptor 1 (encoded by the CSF1R gene on chromosome 5q32) have been described to be causative for the disease [1]. The CSF1R plays an important role in microglial functioning. It is a cell surface receptor for the cytokine CSF-1 that regulates mononuclear phagocytic cells, including microglia of the central nervous system [4]. Microglial dysfunction is therefore believed to play a central role in the pathogenesis of HDLS.

Here we report on a case of a 29 year-old female suffering from hereditary diffuse leukodystrophy with spheroids, which was confirmed by a stereotactic biopsy as well as genetic testing. We found a mutation of the colony stimulating factor 1 receptor (CSF1R) at c.2381 T > C (p.I794T) that was previously described in one patient to be causative for HDLS. Notably, a Medline search with the two most conspicuous features of this case suggested the diagnosis of HDLS before biopsy or genetic testing were done.

## Case presentation

We report on a 29 year-old female patient who presented with a slow onset of gait disturbance, spasticity, ataxia, dysarthria and cognitive decline developing over a peroid of just 4 months. The patient formerly had been healthy and the family history was unremarkable with the exception of her grandfather who had died from Alzheimer’s disease. In the clinical examination she showed anisocoria (L > R), a discrete left facial nerve palsy, a spasticity with dyskinetic movements of the left upper and lower limb, brisk tendon reflexes at both knees and ankles with a positive Babinski sign on both sides and an ataxic gait with small steps. Furthermore she displayed a staccato speech with dystonic movements of the mandibular muscles without any signs of aphasia. She was apraxic throughout the examination when being asked to follow basic instructions. Otherwise the general physical and neurological examination as well as the medical and developmental history were unremarkable with no severe neonatal or infantile illnesses. There was no history of medication or substance abuse.

The following investigations were all normal: Routine blood analysis, search for infectious diseases (HIV, Borrelia burgdorferi, Treponema pallidum, Hepatitis B/C, HSV, VZV), thyroid function tests (TSH, fT3/4, TSH receptor antibodies, thyroperoxidase antibodies, thyroglobulin antibodies), metabolic investigations (α-galactosidase A, very long chain fatty acids, arylsulfatase A, beta- galactocerebrosidase, hexosaminidase A/B, folate, vitamine B12, methylmalonic acid, homocystein, coeruloplasmin), vasculitis screen (ANA, ANCA, RF, Cardiolipin) as well as the cerebral angiography and cerebrospinal fluid analyses (glucose, proteins, cells). The electroneuromyography and the electroencephalogram did not reveal any specific pathological findings. Also the whole-body PET-CT scan was normal.

### Neuroradiological findings

**Fig. 1:**
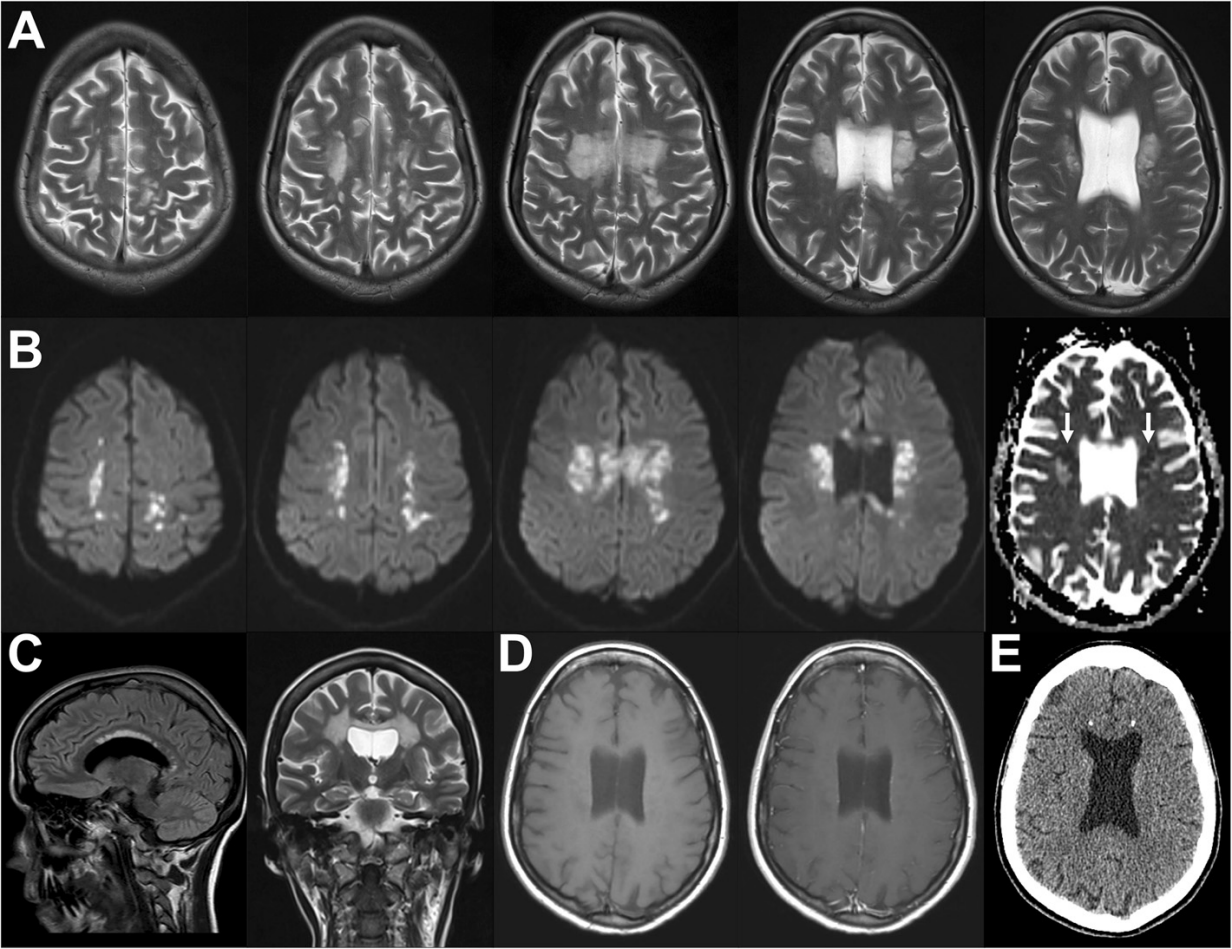
Neuroradiological findings. **a** MRI shows confluent hyperintense changes of the white matter on T2-weighted images, predominantly involving the pyramidal tract. **b** All areas displayed restricted diffusion, last image in row shows decreased values on ADC (white arrows). **c** Involvement of the corpus callosum on sagittal FLAIR (left) and coronal T2-weighted images (right). **d** Lack of contrast enhancement on T1-weighted images before (left) and after application of gadolinium (right). **e** On computed tomography two spots of calcification are seen in both frontal lobes

**Fig. 2:**
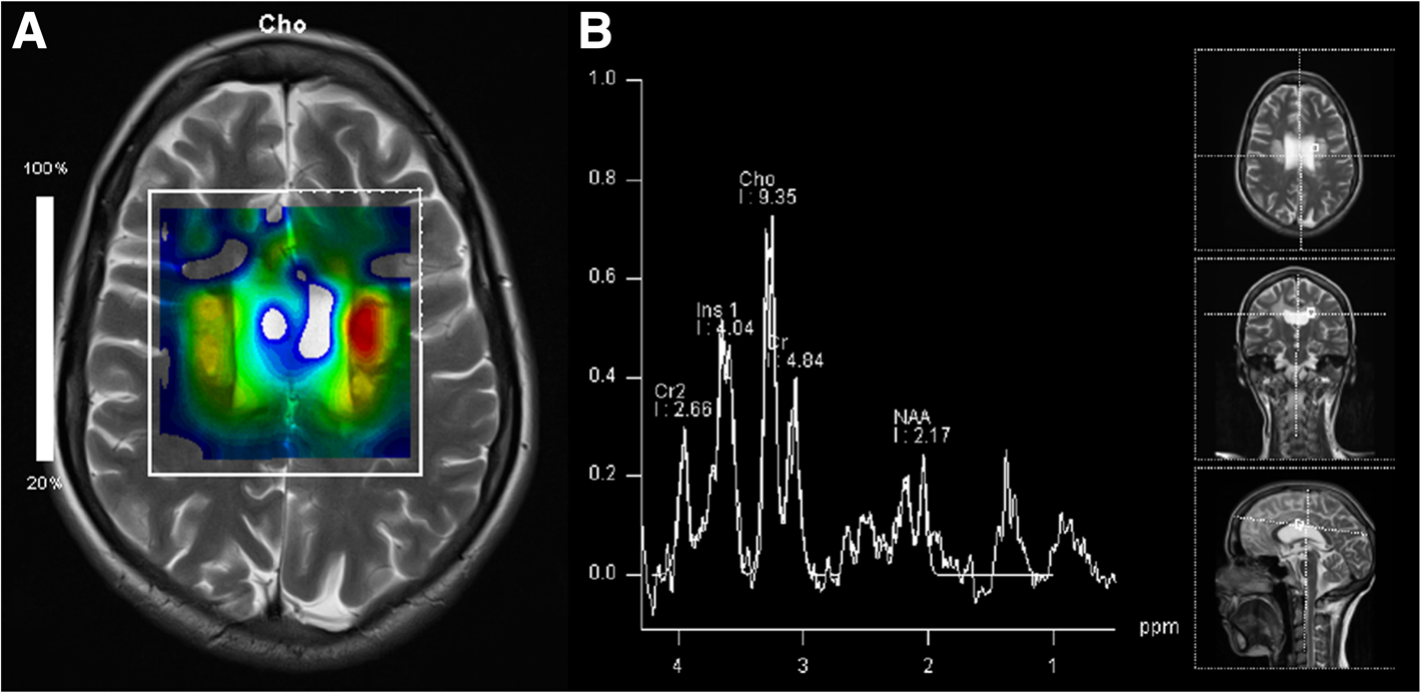
MR spectroscopy. **a** Chemical-shift-imaging shows a choline “hot spot” (yellow and red) within the lesions. **b** MR spectrum in the left lesion indicates not only increased choline level, but also decreased NAA- and creatine-level, while no lactate- or lipid-peak was detectable

MRI showed well-circumscribed, spotted and confluent hyperintensities on T2- and FLAIR-weighted images symmetrically in the periventricular white matter and in the corpus callosum (Fig. 1a + c). Those changes included and extended along the corticospinal tracts and the subcortical white matter sparing the U-fibers. On T1-weighted images, lesions were slightly hypo- to hyperintense, and after intravenous gadolinium application, no contrast enhancement was seen (Fig. 1d). Lesions showed strikingly restricted diffusion with decreased ADC-values (Fig. 1b). DSC perfusion did not reveal any changes of CBF or CBV (not shown). Compared with normal appearing white matter, 1H-MR-spectroscopy (TE 35 ms) of the affected areas revealed an unspecific pattern with an increased choline-peak (131 %), a decreased peak of N-acetylaspartate (31 %), and a decreased creatine-peak (73 %), while no lactate- or lipid-peaks were found (Fig. 2). On CT, those areas showed hypodensity, and small calcifications were found within the lesions symmetrically in the frontal white matter adjacent to the callosal truncus and in the callosal splenium (Fig. 1e). Conventional angiography of the extra- and intracranial vessel was unremarkable and excluded signs of vasculitis (not shown).

As all examinations at that point were not clearly indicative for a specific disease we referred the patient for a stereotactic brain biopsy.

### Neuropathologic findings

**Fig. 3:**
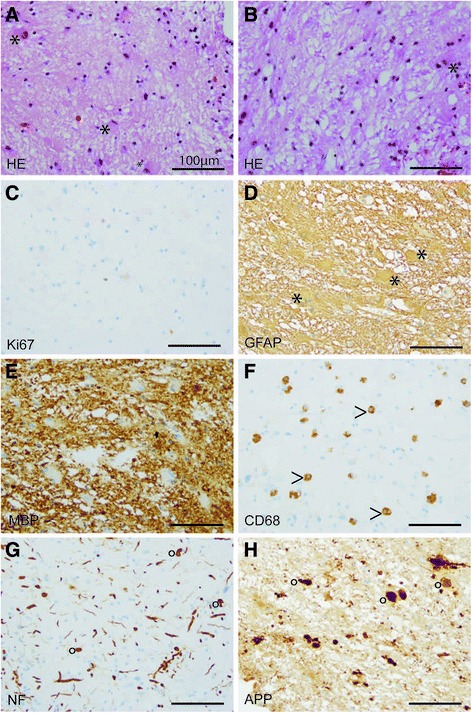
Light microscopy. Histological (**a**, **b**) and immunohistochemical (**c**-**h**) stainings. Biopsy specimens showing many reactive astrocytes (asterisks, **a**, **b**) on a loosened fibrillary CNS matrix. No proliferative activity is observed (**c**). Reactive astrocytes are strongly GFAP-positive (asterisk, **d**). MBP-positive myelin sheats are largely preserved (**e**). Numerous CD68-positive macrophages (arrow head, **f**) infiltrating CNS tissue were observed. NF-positive swollen axons (**g**) as well as NF- and APP-positive axonal spheroids (circle, **g**, **h**). (scale bares = 100 μm)

**Fig. 4:**
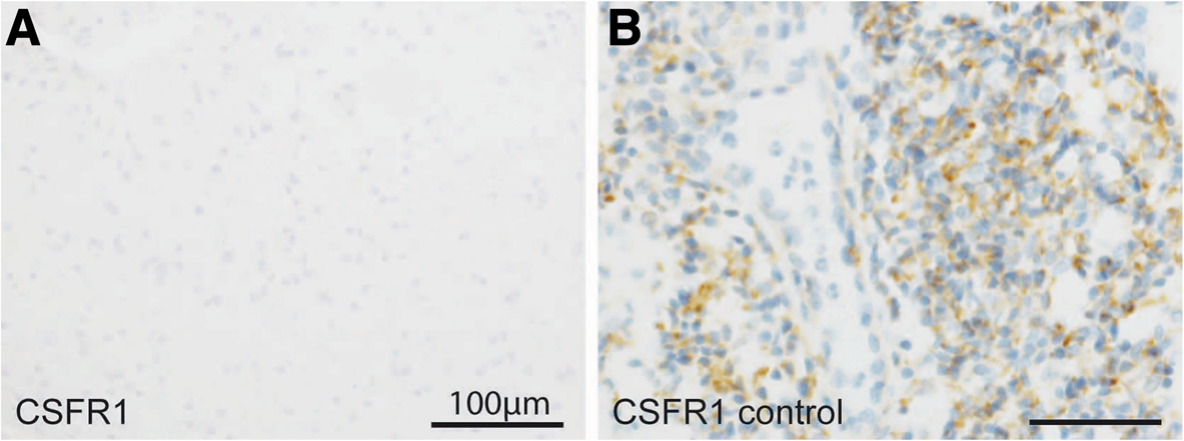
Immunohistochemistry for CSFR1. CSFR1 immunohistochemistry of a stereotactic biopsy sample (**a**) and on tonsil (**b**), the latter serving as positive control tissue (scale bares = 100 μm). Despite proper control, no CSFR1-signal was detected in our patient

**Fig. 5:**
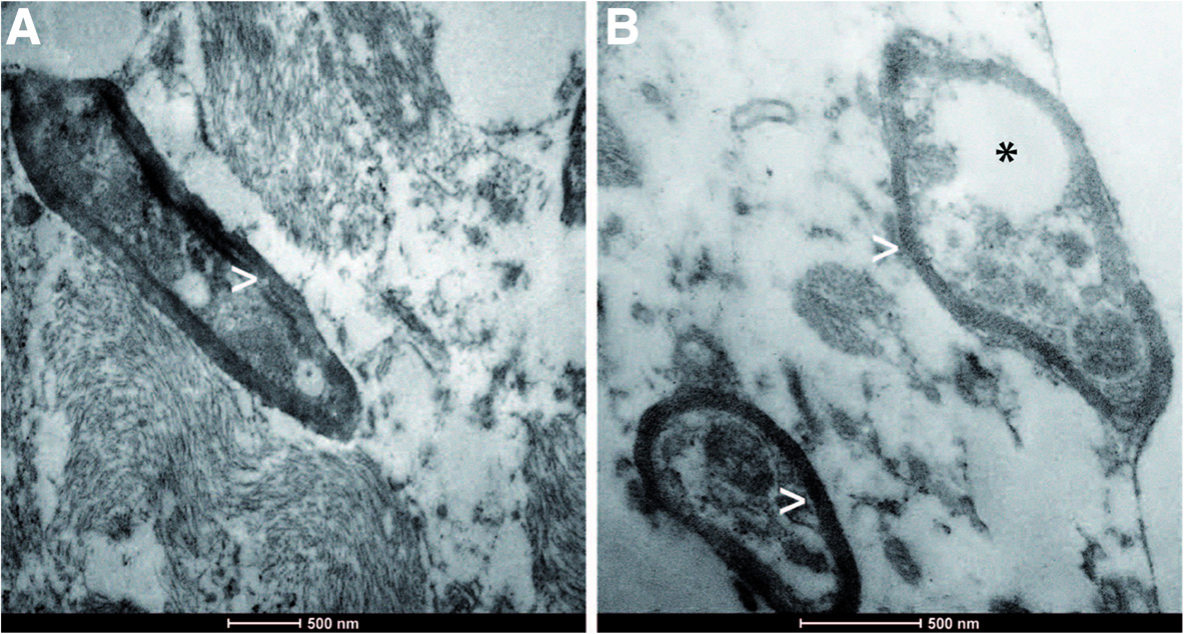
Electron microscopy. Electron microscopy showing brain tissue with regularly myelinated axons (arrow heads, **a** + **b**). Intra-axonal globular inclusions (asterisk, **b**) were observed reflecting the pathological findings of axon spheroids seen in the light microscopic examination

Biopsy specimens from the stereotactic biopsy showed CNS tissue with signs of reactive gliosis and extensive infiltration with CD68-positive macrophages. Furthermore, many axonal spheroids were seen which stained positive for neurofilament (NF) and amyloid precursor protein (APP). The Ki67 proliferation rate was not increased. The myelin was mainly preserved (Fig. 3). In summary, we did not observe hints for an inflammatory or a primary demyelinating disease. JC-virus in situ hybridization to exclude the possibility of a progressive multifocal encephalopathy was negative. The brain biopsy specimens were also negative for CMV, EBV, HSV1/2, as well as VZV. CSF1R immunohistochemistry was negative while control samples revealed positive staining results (Fig. 4). Electron microscopy revealed regularly myelinated axons however several axonal globular inclusions (Fig. 5).

The initial descriptive neuropathological diagnosis was leukoencephalopathy with extensive axonal spheroids. Following the algorithm approach to adult-onset leukodystrophies of Alturkustani and colleagues, we finally favored an adult-onset leukoencephalopathy/leukodystrophy with axonal spheroids (ALAS) related to CSF1R mutation [7]. As other adult leukoencephalopathies/leukodystrophies do usually not go along with the formation of axonal spheroids, our main differential diagnosis was Nasu-Hakola’s disease related to TREM2/DAP12 mutations, however since bone cysts and lipodystrophy were not reported in our patient, this diagnosis was rather unlikely.

### Genetic testing

The genetic testing revealed a heterozygous mutation in exon 18 (c.2381 T > C) of the CSF1R-gene leading to a change in the amino acid sequence (p.I794T). This mutation has been previously described in a family with clinically diagnosed atypical CADASIL syndrome [1]. As our patient’s parents were not available for testing and her family history was not fully known, it is not possible to confirm that this mutation is de novo. Nonetheless, there was no family history of significance reported.

## Discussion

Here we report on a patient showing severe motor dysfunction with gait disturbance, spasticity and ataxia. Moreover, she suffered from progressive cognitive impairment. One of the cerebral lesions showing a hyperintense signal on T2 sequences and restricted diffusion was biopsied. The histology revealed the diagnosis of HDLS, which was confirmed by genetic testing.

The clinical presentation of this patient seems to be rather typical for HDLS, matching with previous reports. Our findings of white matter lesions on T2 sequences without contrast enhancement on T1 are characteristic features in patients with HDLS. Involvement of the corticospinal tract and the corpus callosum, as seen in our patient has been described [6]. However, little is known about other radiological features of HDLS. On a CT scan we found two spots of calcification in both frontal lobes (Fig. 1c). This has previously been described in one patient only [8]. Still, the significance of this finding is unclear. Moreover, our patient showed areas of markedly restricted diffusion. Symmetric supra-tentorial white matter lesions with restricted diffusion and metabolite abnormalities in magnetic resonance proton spectroscopy are also seen in other cerebral pathologies including posterior reversible encephalopathy syndrome (PRES), toxic encephalopathies, or neuronal intranuclear inclusion disease [9–11]. In contrast to previous reports and without exceptions, all areas altered on T2 sequences showed restricted diffusion. Notably, the overlapping changes on T2 and DWI showed a patchy pattern in subcortical areas, but were clearly circumscribed in the deeper white matter (Fig. 1a and b). Large areas of the white matter appeared to be unaffected. Our patient was rapidly progressing and had to be referred to a nursery home within less than 6 months from diagnosis. She had lost the ability to communicate and was not able to stand or walk anymore. Maybe the rather circumscribed MRI pattern and especially the striking diffusion restriction is indicative for a higher acuteness and thus a more unfavorable course of the disease. A more diffuse and patchy pattern with less prominent lesions of restricted diffusion seems to be more common in the cases reported so far [12]. In this case series also two asymptomatic patients, 29 and 69 years of age, carrying a CSF1R mutation were examined. Interestingly, both showed T2 alterations but lacked lesions with restricted diffusion.

Regarding MR spectroscopy we found a markedly increased level of choline while N-acetylaspartate (NAA) was reduced. These changes have been recently reported in three patients with HDLS [12]. We did not find clearly increased levels of myo-inositol or lactate. The increase of choline usually is interpreted as a sign of demyelination and the NAA decrease is most likely the result of axonal damage. Both phenomena have been described in patients with HDLS. As this spectroscopic pattern is found in several leukoencephalopathies MR spectroscopy does not seem to be helpful in distinguishing the underlying diagnosis in these patients. Notably, we did not find demyelination in the biopsy, challenging the increased choline level as a sign of demyelination (Figs. 3, 4 and 5). Another possible source of elevated choline levels in our patient might be the extensive infiltration of macrophages [13]. The negative immunohistochemistry for CSF1R in our patient supports the hypothesis that haploinsufficiency may play a role in HDLS [14]. Konno and colleagues also found markedly reduced CSF1R protein in Western Blots of brain tissue from two HDLS patients.

Aside from the impressive clinical symptoms the MRI showed alterations of the white matter on T2 sequences with striking areas of restricted diffusion. As this diffusion restriction on the basis of a leukoencephalopathy was so impressive we searched for these terms in Medline and got HDLS as one of the first results. Nonetheless, the stereotactic biopsy was performed and corroborated the diagnosis. Finally, the histological diagnosis was confirmed by the genetic testing.

## Conclusion

In conclusion, this case points out at two important features of HDLS being spotted and/or confluent leukoencephalopathy with areas of restricted diffusion. This might help to identify more patients with this underdiagnosed disease. Moreover, the clinical course in our patient from symptom onset and diagnosis was unfavorable and raises the question whether the pronounced areas of restricted diffusion are indicative of a more acute progression of the disease.

### Consent

Written informed consent was obtained from the patient for publication of this Case report and any accompanying images. A copy of the written consent is available for review by the Editor of this journal.

## Abbreviations

ADC: Apparent diffusion coefficient
ANA: Anti-nuclear antibodies
ANCA: Anti-neutrophil cytoplasmic antibodies
APP: Amyloid precursor protein
CADASIL: Cerebral autosomal-dominant arteriopathy with subcortical infarcts and leukoencephalopathy
CBF: Cerebral blood flow
CBV: Cerebral blood volume
CBS: Corticobasal Syndrome
CD68: Cluster of Differentiation 68 (macrophage marker)
CMV: Cytomegalovirus
CSF1R: Colony stimulating factor 1 receptor
DSC: Dynamic-susceptibility contrast
EBV: Ebstein barr virus
FTD: Frontotemporal dementia
GFAP: Glial fibrillary acidic protein
HE: Hematoxylin and eosin
HIV: Human immunodeficiency virus
HSV: Herpes simplex virus
Ki67: Kiel 67 (proliferation marker)
MBP: Myelin basic protein
MS: Multiple sclerosis
NF: Neurofilament
PET: Positron emission tomography
PRES: Posterior reversible encephalopathy syndrome
RF: Rheumatic factor
TSH: Thyroid stimulating hormone
VZV: Varicella zoster virus
